# Supplementary material for: Validation of the shortened version of the Canine Behavioral Assessment and Research Questionnaire (C-BARQ) using participants from the Dog Aging Project
Source: PLoS One. 2024 Apr 11;19(4):e0299973. doi: 10.1371/journal.pone.0299973 (PMC11008875; doi:10.1371/journal.pone.0299973)
Supplement: Attachment — Submitted filename: Canine Behavioral Assessment and Research Questionnaire (C-BARQ) using participants from the Dog Aging Project.pdf [file pone.0299973.s002.pdf]

This work is very interesting and also very hard as answering to surveys require a lot of time and effort from the owners, and I admire this type of work very much.

A few questions arose while going over the work, which I will need some clarity on:

Do you have the demographics of the owners who filled out the questionnaire? Such as gender, age, level of education and social class?

Do you believe the gender or other characteristics of the owners who responded to the questionnaire could influence their responses? On that same note, I believe if table 1 contained demographic information about the owners, it could reduce possible biases and/or raise new questions.

Do you know if the same owners responded to both the long and short questionnaires and if they responded at the same time? I raise this question because the questionnaires are long which could influence the effort the owners put into responding the question towards the end of the survey.

On the discussion, I felt the point of the possible reason on aggressiveness was missed. Could it be because the questions are ambiguous and hard to interpret which can be answered in different ways? I also believe it’s relevant to discuss how answers regarding gender and size/type of breed can influence on the animal’s aggressiveness? Is there any type of association already described?
