# Supplementary material for: Validation of the shortened version of the Canine Behavioral Assessment and Research Questionnaire (C-BARQ) using participants from the Dog Aging Project
Source: PLoS One. 2024 Apr 11;19(4):e0299973. doi: 10.1371/journal.pone.0299973 (PMC11008875; doi:10.1371/journal.pone.0299973)
Supplement: Attachment — Submitted filename: Response to reviewers.docx [file pone.0299973.s003.docx]

1. Please ensure that your manuscript meets PLOS ONE's style requirements, including those for file naming. The PLOS ONE style templates can be found at 
<https://journals.plos.org/plosone/s/file?id=wjVg/PLOSOne_formatting_sample_main_body.pdf> and 
<https://journals.plos.org/plosone/s/file?id=ba62/PLOSOne_formatting_sample_title_authors_affiliations.pdf>

*I have updated the headings, file names, etc. to adhere to the style requirements.*

2. You indicated that ethical approval was not necessary for your study. We understand that the framework for ethical oversight requirements for studies of this type may differ depending on the setting and we would appreciate some further clarification regarding your research. Could you please provide further details on why your study is exempt from the need for approval and confirmation from your institutional review board or research ethics committee (e.g., in the form of a letter or email correspondence) that ethics review was not necessary for this study? Please include a copy of the correspondence as an ""Other"" file.

*University of Washington’s IRB reviewed the study proposal and determined the study qualifies for Exemption, Category 2 – research involving use of survey procedures and interview procedures. I have requested a letter from my colleague at UW who initiated the IRB process there and will upload that confirmation as an “other” file as soon as I receive it.*

3. Note from Emily Chenette, Editor in Chief of PLOS ONE, and Iain Hrynaszkiewicz, Director of Open Research Solutions at PLOS: Did you know that depositing data in a repository is associated with up to a 25% citation advantage (<https://doi.org/10.1371/journal.pone.0230416>)? If you’ve not already done so, consider depositing your raw data in a repository to ensure your work is read, appreciated and cited by the largest possible audience. You’ll also earn an Accessible Data icon on your published paper if you deposit your data in any participating repository (<https://plos.org/open-science/open-data/#accessible-data>).

*All data associated with the Dog Aging Project is curated and open access is available through our website.*

4. We note that the grant information you provided in the ‘Funding Information’ and ‘Financial Disclosure’ sections do not match.

When you resubmit, please ensure that you provide the correct grant numbers for the awards you received for your study in the ‘Funding Information’ section

*We have included the funding support for the entire Dog Aging Project, but this specific research project was not funded. We have included this statement for the Financial Disclosure in our cover letter: “The author(s) received no specific funding for this research project, but data collection for the Dog Aging Project is supported by U19 grant AG057377 from the National Institute on Aging, a part of the National Institutes of Health. In addition, each of the named authors on this manuscript have received salary support from the U19 grant.”*

5. Thank you for stating the following financial disclosure: 
"The funders had no role in study design, data collection and analysis, decision to publish, or preparation of the manuscript."

At this time, please address the following queries:

a) Please clarify the sources of funding (financial or material support) for your study. List the grants or organizations that supported your study, including funding received from your institution.

*This clarification has been included in the Financial Disclosure.*

b) State what role the funders took in the study. If the funders had no role in your study, please state: “The funders had no role in study design, data collection and analysis, decision to publish, or preparation of the manuscript.”

*This statement has been included in the Financial Disclosure.*

c) If any authors received a salary from any of your funders, please state which authors and which funders.

*This has been included in the Financial Disclosure.*

d) If you did not receive any funding for this study, please state: “The authors received no specific funding for this work.”

Please include your amended statements within your cover letter; we will change the online submission form on your behalf.

*We have included the amended statement in the cover letter.*

6. We note that you have included the phrase “data not shown” in your manuscript. Unfortunately, this does not meet our data sharing requirements. PLOS does not permit references to inaccessible data. We require that authors provide all relevant data within the paper, Supporting Information files, or in an acceptable, public repository. Please add a citation to support this phrase or upload the data that corresponds with these findings to a stable repository (such as Figshare or Dryad) and provide and URLs, DOIs, or accession numbers that may be used to access these data. Or, if the data are not a core part of the research being presented in your study, we ask that you remove the phrase that refers to these data.

*This has been removed.*

7. One of the noted authors is a group or consortium [The Dog Aging Project Consortium]. In addition to naming the author group, please list the individual authors and affiliations within this group in the acknowledgments section of your manuscript. Please also indicate clearly a lead author for this group along with a contact email address.

*The consortium does not have a “lead author,” but we do have a consortium email address and that has been included in the acknowledgement section.*

8. Please include your full ethics statement in the ‘Methods’ section of your manuscript file. In your statement, please include the full name of the IRB or ethics committee who approved or waived your study, as well as whether or not you obtained informed written or verbal consent. If consent was waived for your study, please include this information in your statement as well.

*This has been added.*

9. Please review your reference list to ensure that it is complete and correct. If you have cited papers that have been retracted, please include the rationale for doing so in the manuscript text, or remove these references and replace them with relevant current references. Any changes to the reference list should be mentioned in the rebuttal letter that accompanies your revised manuscript. If you need to cite a retracted article, indicate the article’s retracted status in the References list and also include a citation and full reference for the retraction notice.

*The reference list has been reviewed and is complete and correct.*

**REVIEWER #1**:
This work is very interesting and also very hard as answering to surveys require a lot of time and effort from the owners, and I admire this type of work very much.

*Thank you so much for this comment. We are excited to be able to present some of the work we are doing with data collected at the Dog Aging Project.*

A few questions arose while going over the work, which I will need some clarity on:
Do you have the demographics of the owners who filled out the questionnaire? Such as gender,
age, level of education and social class?

*In order to adhere to our IRB exemption, we only collect minimal information about the dog owners. That information includes age categories, highest level of education, and annual income categories. We do not collect any information about sex or gender and owners are able to select “prefer not to answer” for the income question.*

Do you believe the gender or other characteristics of the owners who responded to the
questionnaire could influence their responses? On that same note, I believe if table 1 contained
demographic information about the owners, it could reduce possible biases and/or raise new
questions.

*We really appreciate this suggestion. We have added owner demographic information (age, education level, and income) to table 1. Due to the limitations regarding human-level data collection mentioned above, the owner information is categorized and thus would likely limit our ability to infer a great deal from it. However, this could be hypothesis generating for future studies.*

Do you know if the same owners responded to both the long and short questionnaires and if
they responded at the same time? I raise this question because the questionnaires are long
which could influence the effort the owners put into responding the question towards the end
of the survey.

*Only owners who completed both versions of the survey were included in this analysis. The short version of the survey was completed first in July of 2020 as part of the HLES survey. The long version of the survey was completed in August of 2020 as a standalone survey. Thus, even though the survey itself was longer, there were no other surveys being completed at the same time. Information about the timing of the survey completions is included in the methods subsection entitled “Study population.”*

On the discussion, I felt the point of the possible reason on aggressiveness was missed. Could it
be because the questions are ambiguous and hard to interpret which can be answered in
different ways? I also believe it’s relevant to discuss how answers regarding gender and size/type
of breed can influence on the animal’s aggressiveness? Is there any type of association already
described?

*We limited the discussion about the specific results of the surveys as our main purpose was to validate that similar responses can be obtained from owners of dogs with multiple temperament types using either version of the C-BARQ survey (long or short). We felt that an in depth discussion about the causes of aggressiveness in dogs – or really causes of why owners report aggressiveness in dogs – would detract from that purpose. We do agree that there can be misinterpretation of questions, but feel that we have shown that regardless of the accuracy, owners report similar responses using both survey types.*

REVIEWER #2:
I think the analysis was performed rigorously and explained in details. I am concerned about the 60% response rate. While the authors did a great job of mentioning it in the limitations, I think more information can be included about why they think this happened, how it may impact the results, and how the authors are going to mitigate the effects.

*Thank you so much for taking the time to review this manuscript. We appreciate your effort and comments. We feel that the 60% response rate at the height of the pandemic and pre-vaccine was acceptable, but agree that we needed to increase the discussion about that point. The discussion now reads: “Second, while the response rate at nearly 60% was acceptable, particularly given the timing of the survey request in relation to world events, the potential for non-response bias is worth mentioning. For instance, it could be that participants that were not able to complete the long version of the C-BARQ survey were those who were no longer under quarantine restrictions in place due to the pandemic and thus their dogs may have been experiencing a shift in their daily routine that could have led to less agreement between survey responses than within the population who was able to complete both surveys.”*

Please also review the article generally for flow, spelling, grammar, etc. now that it has been a few weeks since it was originally submitted.

Thank you – we hope that it reads smoothly, but are happy to adjust as needed!
